# Smart Exhaust Analytics: A Sensor-Based Way to Identify the Types of Engines Based on the Composition of Exhaust Gas

**DOI:** 10.3390/s26092863

**Published:** 2026-05-03

**Authors:** Dharmendra Kumar, Vibha Jain, Ashutosh Mishra, Rakesh Shrestha, Navin Singh Rajput

**Affiliations:** 1 Department of Electronics Engineering, Indian Institute of Technology (BHU), Varanasi 221005, India; dharmendrakumar.rs.ece19@iitbhu.ac.in; 2 Department of Computer Science and Engineering, Thapar Institute of Engineering and Technology, Patiala 147004, India; vibha.jain@thapar.edu; 3 Department of Electrical and Electronics Engineering, Birla Institute of Technology and Science Pilani-Dubai, Dubai 345055, United Arab Emirates; 4 Smart Automation and Cyber Resilience (SACR) Unit, Research Institute of Sweden (RISE), 114 86 Stockholm, Sweden

**Keywords:** electronic nose (E-nose), hazardous gas estimation, engine classification, MQ-series sensors, intelligent systems

## Abstract

Classification of vehicle engines using the chemical composition of the exhaust from these engines can be used to identify the engine’s design and verify compliance with environmental regulations through the vehicle’s emissions. This paper describes a method to identify the type of vehicles using machine learning (ML), where low-cost MQ series sensors measure the gases and particle emissions from a vehicle exhaust system, while simultaneously collecting and measuring the vehicle’s temperature and humidity levels. A custom-designed multi-sensor exhaust sensing module is employed to capture real-time exhaust emissions prior to entering the atmosphere. Exhaust samples are collected from vehicles representing three major engine categories: petrol, diesel, and compressed natural gas (CNG). In addition, fresh air samples are collected as a baseline environmental reference for comparison. All exhaust measurements are collected under controlled and consistent engine operating conditions to ensure comparable emission profiling across vehicle classes. To ensure consistent combustion-based emission profiling, this study focuses on conventional fuel-powered vehicles. MQ-series gas sensors are sensitive to combustion by-products emitted during engine operation, such as carbon monoxide (CO), hydrocarbons (HC), while also exhibiting cross-sensitivity to other gaseous components present in exhaust mixtures. Nevertheless, the proposed system performs pattern-based classification using relative sensor response signatures. Standardization of data is achieved through z-score normalization. The best models developed (based on three separate experimental designs) are trained and validated using six supervised machine learning algorithms such as Logistic Regression, Support Vector Machine (RBF), k-Nearest Neighbors, Random Forest, Gradient Boosting Decision Tree, and XGBoost and are compared against one another. Evaluation of the tested algorithms using various evaluation metrics demonstrated that ensemble models outperformed all other algorithms, achieving the highest accuracy of 99.5%. Furthermore, noise analysis confirms that the proposed solution maintains high classification accuracy (more than 89%) even under substantial sensor perturbations mimicking the real-world deployment. The solution proposed below illustrates how using gas sensors and advanced algorithms can provide accurate exhaust identification and identify engines in real-time.

## 1. Introduction

Car exhaust, a leading source of urban air pollution, emits a complex mixture of nitrogenous species, hydrocarbons, carbon monoxide, and hydrogen, as well as particulate and volatile organic compounds. Car exhaust emissions should be analyzed to provide a comprehensive understanding of engine performance, support diagnosis and correction of combustion inefficiencies, and monitor compliance with government regulations and standards [1]. Transportation in urban states plays a critical role in air pollution, releasing hydroxides, carbon monoxide, hydrogen, methane, volatile organic compounds, and particulate-bound pollutants [2]. A study examined seasonal fluctuations between 2015 to 2019, to observe PM10 aerosols and corresponding trace metal concentrations in the urban city of Raipur, India. They studied eighteen trace elements (Al, Ba, Ca, Cr, As, Mn, Sb, Se, Sr, Cu, Fe, Ga, Mg, Ni, Ti, V, Pb, Zn) and (EC and OC) by atomic absorption spectroscopy and thermal optical transmittance [3,4]. Although the traditional exhaust measurement tools offer gas quantification accuracy, such systems are often large, confined in laboratories, and too expensive to be used on a large scale [5]. The emergence of low-cost metal–oxide semiconductor (MOS) gas sensors, embedded microcontrollers, and portable data logging technologies offers a promising alternative for real-time, in-field exhaust analysis [6,7]. To classify engines, diagnose emissions, and comply with environmental regulations, it is crucial to accurately measure and describe these emissions. However, traditional exhaust analysis systems are often large, only work in laboratories, and are expensive, making them less helpful in monitoring different vehicle conditions in the field or in real-time. Recent improvements in low-cost gas sensors, wireless embedded platforms, and edge-level data-acquisition technologies have enabled the development of portable, scalable diagnostic tools that can collect multi-pollutant signatures directly from the source.

Traditional on-board diagnostic (OBD) systems can detect emission-related issues; however, they may not provide a complete classification of engine types and situations based on emission signatures [8,9]. This gap needs the development of specialized algorithms capable of identifying vehicular engines based on their exhaust emission patterns [10]. Motorcycle emissions and fuel consumption in a rising Asian city are evaluated using on-road exhaust-emission and fuel-consumption models designed for traffic micro-simulation [11]. This motorbike onboard measuring system is developed to measure and continuously record on-road driving data, such as speed-time profile, exhaust emissions, and fuel consumption per second [12].

Integrating several inexpensive gas sensors into a single vehicle exhaust measurement system is a nontrivial problem, mainly because it increases the complexity of multiplexing analog signals and calibrating sensors, compensating for environmental changes such as humidity and temperature, synchronizing timestamps among the sensors, and obtaining consistent, accurate readings as the engine load or dynamics change [13]. Additionally, accurately characterizing the exhaust often requires simultaneous detection of multiple gases from the multi-engine family (petrol, diesel, CNG) and fresh air, which increases the challenge of routing, controlling, and managing power on resource-limited embedded hardware [14,15]. To address these issues, this work develops an integrated sensing and machine-learning system to classify vehicle engine type based on exhaust gas signatures while discussing issues such as class imbalance and the generalization performance of supervised learning models.

Although MQ-series MOS sensors have several strengths in terms of cost and portability, they have a number of inherent limitations that should be taken into account during the interpretation of exhaust measurements. MQ sensors are cross-sensitive due to non-specific gas adsorption and can be used for pattern recognition of gases, but not for selective quantification of gases. Low chemical selectivity is a feature of these types of sensors, i.e., strong cross-sensitivity, when sensing, these sensors can respond to multiple reducing gases with a similar change in resistance. Moreover, MOS sensors also respond to environmental factors like temperature and humidity that can also lead to a drift in the base and inconsistency in measurements with time. stability may also be affected by thermal drift caused by the operation of the heater and long-term sensor aging. MQ sensors, therefore, are more applicable in qualitative pattern-recognition systems of home-based electronic noses and not in the quantification of controlled pollutants. Conversely, this is compared to technology that is considered well-developed, like Portable Emissions Measurement Systems (PEMS) and Fourier Transform Infrared (FTIR) analyzers, which offer a highly selective and calibrated analysis of regulated pollutants, including CO, hydrocarbons, and particulate matter [16]. However, such instrumentation is typically expensive, bulky, and unsuitable for portable or large-scale distributed monitoring scenarios.

To address some of the issues with traditional laboratory-based exhaust measurement systems, this study develops a portable and field-deployable framework for real-time multi-pollutant exhaust emissions. The proposed battery-powered multi-sensor embedded platform enables synchronized multi-gas data acquisition under controlled sampling conditions, facilitating the creation of a high-resolution dataset of exhaust signatures for multi-class engine classification. The suggested experimental design is particularly constrained to stationary (standby) vehicle operation, where sampling of exhaust is conducted in regard to stable idle operation. The sensor responses gained are used to recognize patterns of exhaust signatures so as to classify engines. The results are therefore not expected to portray dynamic or on-road driving conditions.

The major contributions of this study are as follows:

- Development of a low-cost, battery-powered, multi-sensor embedded platform for real-world exhaust gas acquisition that features eight MQ-series gas sensors, an environmental sensing unit, an RTC module, an analog multiplexer, a microSD interface, and an ESP8266 microcontroller.
- A curated exhaust-gas dataset consists of 3009 labeled samples with three engine type classes, such as Petrol, Diesel and CNG include a baseline of fresh air. These samples are collected under controlled exposure conditions and preprocessed for reproducible machine-learning experimentation.
- Developing a comparative assessment system of the supervised machine learning methods, such as: linear, kernel-based, instance-based, and ensemble.

The subsequent sections elaborate on the work associated, system architecture, embedded circuit design, data acquisition, dataset description and experimental set-up employed in this study.

## 2. Related Works

The increasing demand for portable and low-cost vehicle emission monitoring has stimulated extensive research into compact gas sensors, embedded platforms, and data-driven exhaust analysis methods. Spinelle et al. [17] demonstrated the potential of miniaturized gas sensors for urban air-quality monitoring while also highlighting critical challenges, such as calibration complexity, drift, and environmental sensitivity, that limit standalone reliability. Kumar et al. [18] emphasized the need for scalable, field-deployable sensing networks capable of capturing spatiotemporal pollution dynamics at the city scale.

Regulatory developments, particularly the evolution of Euro emission standards, have further accelerated advancements in emission instrumentation and modeling. The introduction of particle number (PN) limits under Euro 6 required controlled dilution systems and chemical speciation techniques to ensure measurement accuracy [19]. Additionally, studies on vehicular particulate composition indicate that motorcycle emissions are predominantly composed of organic carbon fractions [20]. Long-term monitoring data from the UK-AIR report substantial reductions in CO and HC emissions from Euro 3 to Euro 6 vehicles; however, persistent discrepancies between laboratory and real-world NOx emissions underscored the need for on-road validation systems such as Portable Emissions Measurement Systems (PEMS) [21]. Studies on low-cost metal-oxide semiconductor (MOS) sensors have demonstrated their ability to detect combustion by-products, such as carbon monoxide (CO), hydrocarbons (HC), and methane, with response characteristics suitable for exhaust monitoring applications. Badruddin [22] highlighted that while MOS sensors provide rapid response times, their reliability depends on proper heater stabilization and environmental compensation to mitigate drift and temperature–humidity effects. Complementing this, Koc and Madarász [23] demonstrated the feasibility of integrating MQ-series sensors with ESP-based microcontrollers to develop compact, multi-sensor air-quality monitoring platforms, thereby validating the practicality of low-cost embedded sensing architectures for real-time environmental analysis. Environmental influences and calibration constraints remain central challenges in MOS-based sensing. Szczurek et al. [24] demonstrated that temperature and humidity significantly affect sensor response stability, underscoring the need for environmental compensation mechanisms. A CNT–TiO_2_ nanowire-based E-nose system has been reported for VOC detection, demonstrating enhanced sensitivity and reduced response time through temperature modulation and feature extraction (DWT, PCA), achieving up to 97.5% classification accuracy using SVM [25]. Similarly, Gerboles et al. [26] highlighted the importance of calibration strategies and reference alignment when deploying MOS sensors in automotive exhaust environments. From an instrumentation perspective, portable exhaust monitoring architectures have been explored using compact chamber-based and duct-integrated sampling systems, as evaluated by Seshadri [5], reinforcing the feasibility of field-deployable emission sensing platforms. Embedded implementations using low-cost microcontrollers, such as the ESP8266, have further enabled real-time wireless monitoring systems, as demonstrated by Saad and Al-Hiary [27]. Efficient multi-sensor integration techniques, including time-division multiplexing for analog signal acquisition, have also been reported to reduce hardware complexity while maintaining scalability [28]. Regarding exhaust classification using inexpensive semiconductor sensors, Wang and Zhang reported that MQ-series sensors can effectively distinguish engine categories based on emission signatures [29,30]. These studies collectively support the feasibility of low-cost, embedded, multi-sensor architectures for qualitative exhaust-gas analysis, while also underscoring the importance of calibration, environmental compensation, and robust system integration.

Together, these studies illustrate the concept, challenges, and design considerations associated with developing compact, low-cost multi-gas exhaust sensor systems. Prior work has shown the usefulness of using MOS sensors and embedded controllers to monitor indoor air quality. It has identified a continuing need to develop an integrated, battery-operated, portable, time-synchronized, multi-sensor embedded architecture with environmental corrections and high-frequency data logging. The focus of the present work is the profile of the controller architecture employed and the results produced.

## 3. Proposed Methodology

**Figure 1:**
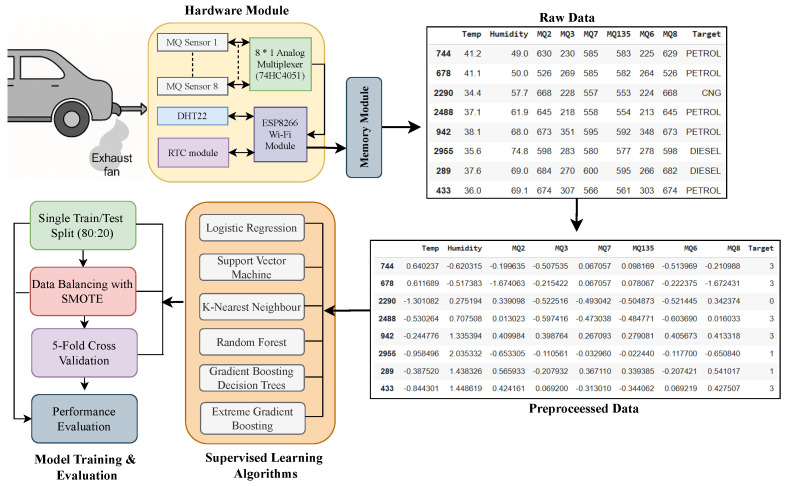
Detailed end-to-end system framework for exhaust-gas based engine classification.

The proposed study follows a structured end-to-end workflow for vehicle engine classification based on exhaust gas signatures. Exhaust samples are collected from petrol, diesel, and CNG-powered vehicles under controlled steady-state operating conditions. In addition, fresh air measurements are used as baseline references. An application-specific sensing module, with MQ-series metal-oxide semiconductor gas sensors and a DHT22 environmental sensor, measures combustion-related gas signatures, ambient temperature, and humidity. The sensor data recorded are checked by examining their completeness and consistency in the format of distribution. Another step involves the z-score normalization of all features to make the scale comparative across heterogeneous sensor channels. The data is stratified with sample size separation to maintain class distributions, and 80:20 train and test separation was used as the baseline, and five-fold stratified cross-validation was done to evaluate the strength. One of the classes of supervised machine learning models is trained. Class imbalance is addressed using the Synthetic Minority Oversampling Technique (SMOTE), applied exclusively to the training data to prevent information leakage. The test dataset remains completely untouched and contains only real observations. Furthermore, as the dataset is collected across multiple acquisition sessions, SMOTE operates within the local feature space of the training data without explicitly mixing information from unseen test sessions. A class of supervised machine learning models is trained. The general approach used in the study is the multi-stage pipeline comprising exhaust data acquisition, preprocessing, model training, and evaluation. Figure 1 shows the complete end-to-end flow of the proposed framework for exhaust-gas-based engine classification.

### 3.1. Experimental Gas Sampling Chamber: Sealing and Flow Configuration Methodology

#### 3.1.1. Sampling Chamber Design

The gas sampling chamber used in the experiment is made out of a tight-fitting polypropylene plastic container. The chamber is temporary housing that is used to collect exhaust gases before sensing and data collection. The container material has been selected as it is chemically inert, can be readily altered, has a lightweight design, and can be reused in an experimental process. A PVC pipe inlet is provided on one side of the container where the inflow of exhaust gases might have been regulated. An external connection of the inlet pipe to a funnel-shaped collector is placed close to the vehicle exhaust vent. The framework enables the direct arrest of exhaust gases and reduces the wastage through the dispersion of the environment. The chamber volume is selected to guarantee that there is sufficient accumulation and mixing of the gases to ensure that the gas sensors provided become reliable and repeatable in response to indications in data collection.

#### 3.1.2. Installation of Pipes

Installation of the exhaust gas inlet involved drilling a hole in the plastic chamber at the location of the external diameter of the used PVC pipe of 3.8 cm diameter as the sampler. The PVC tube is passed in the opening, but in a manner that the pipe has about 2–3 cm of its length being in the chamber interior. Such a design will make the exhaust gases entering the chamber directed towards the internal sensing area. On the outside, the pipe is attached to a funnel-shaped collector, which takes the exhaust plume of the vehicle exhaust outlet and channels it into the pipe. The funnel enhances the effectiveness of gas trapping and provides uniformity of sampling processes in experiments.

#### 3.1.3. Box Sealing Method

In order to sustain the desired amount of gas in the room and to ensure the undesirable leakage does not occur, the container is sealed with industrial adhesive sealing methods. A lid-container interface adhesive packing tape is applied completely along the perimeter of the lid-container interface after affixing the lid securely on the container with a lid. Several coats of sticky tape are used to strengthen the seal to reduce the chances of gas leaking. Adhesive tape is also used to seal the interface between the PVC pipe and the container wall in order to provide an airtight connection. On the same note, adhesive wrapping is used to fit the joint between the funnel and PVC pipe. The reason behind the choice of this sealing method is that it is simple, cheap, and fast to implement where experimental trials are involved. The chamber has been checked visually before data are collected to make sure that there are no visible gaps or points of leakage.

#### 3.1.4. Procedure of Exhaust Gas Collection

**Figure 2:**
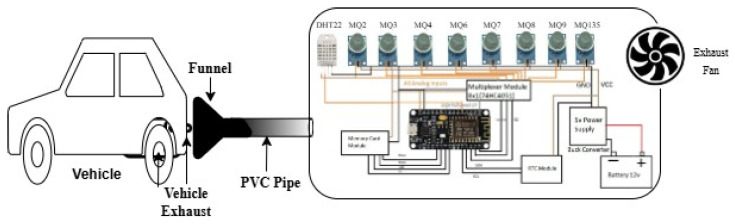
Schematic model of the proposed exhaust gas data acquisition system, illustrating partial-flow sampling of vehicle exhaust using a funnel and PVC pipe into a closed chamber equipped with an MQ-series sensor array, data acquisition module, and supporting electronics for VOC-based emission pattern recognition.

Practically, the funnel opening is placed in proximity to the exhaust outlet of the vehicle, and measurements are made during an experiment range of about 10–15 cm. When the engine is turned on, the funnel trapped the exhaust emitted and directed it into the sealed sampling chamber through the PVC pipe. Gas exhausts drifted inside the chamber and compounded in the sealed chamber. The array of gas sensors installed into the interior chamber reacted to the concentration of gases contained within the exhaust mixture. The sensor data is captured using the data acquisition system made with microcontrollers. This process enabled constant conditions of sensor exposures and provided reproducibility of measurements on a variety of vehicles’ engine types in the collection of data. The Figure 2, shows the proposed experimental setup to be used in the data acquisition of exhaust gases. The system uses a partial-flow sampling method, in which the vehicle exhaust gases are collected with the help of a funnel and passed through a PVC pipe to a closed chamber. The chamber can be fitted with a gas sensor array of the MQ-series with a data acquisition module and complementary electronics. The given configuration allows capturing multi-sensor VOC response patterns that are further used to recognize and classify the pattern of vehicle exhausts. The proposed exhaust gas data acquisition system’s schematic model shows how vehicle exhaust is partially sampled using a funnel and PVC pipe into a closed chamber that has an 8 MQ-series gas sensor array, a data acquisition module, and auxiliary electronics for VOC-based emission pattern recognition.

**Figure 3:**
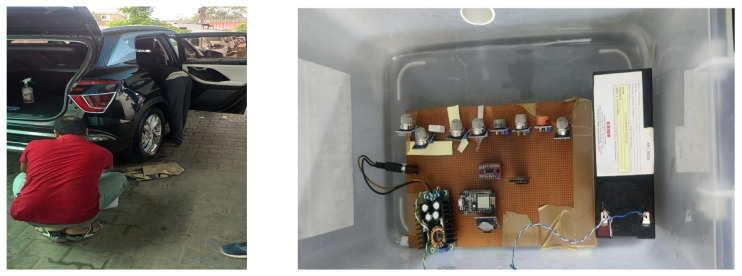
Data acquisition setup for vehicular exhaust monitoring.

The experimental installation is illustrated in Figure 2, and Figure 3 is specially adapted to stationary (standby) vehicle conditions, i.e., the engine is working under the idle or minimum load with no dynamic driving effects. The stationary location of the vehicle is used in data acquisition, with the exhaust gases then sampled through a controlled funnel-PVC pipe setup to a closed sensing chamber. This is used to provide a stable and repeatable sensor exposure, reducing the transient effects of acceleration, turbulence in airflow, and variable load conditions as are found in actual driving conditions. Therefore, the obtained exhaust signatures and subsequent results of classification are only indicative of the behavior of the standby engine, and the present research is not extended to the behavior of the vehicles when they are moving or on the road.

#### 3.1.5. Flow Configuration: Partial Flow

The experimental design is that of a partial-flow exhaust sampling design. In full-flow sampling systems the exhaust stream of the engine is fully directed through a measurement system, which is generally a part of a professional emission test apparatus like dilution tunnels. By comparison, the current experimental arrangement only samples a part of the exhaust plume with a funnel-shaped collection mechanism. The gas sample captured is then taken into the sensing chamber as a pipe to the gas sensors measuring the resulting concentration signatures. As the system does not take up the entire exhaust flow but only a portion of the exhaust plume, the construction is defined as a partial-flow sampling system. This is a common way utilized in a portable gas sensing experiment low priced electronic nose intended to detect the pattern of emissions and not the quantification of emissions imposed by regulation.

## 4. Experimental Setup

The vehicular exhaust data acquisition system developed in this study consists of an integrated embedded architecture comprising eight MQ-series metal-oxide semiconductor gas sensors (MQ2, MQ3, MQ4, MQ6, MQ7, MQ8, MQ9, and MQ135), a DHT22 environmental sensor, a real-time clock (RTC) module, a microSD memory interface, an analog multiplexer, and an ESP-8266 Wi-Fi-enabled microcontroller, all poared through a regulated 5 V supply derived from a 12 V battery using a buck converter. The exhaust sampling system is designed not to force the exhaust plume to interact with the electronics, and the enclosure is not closed to tailpipe exhaust.The metal-oxide sensor heads, or MQ-series sensing components, are not forced to make contact with the exhaust plume, and the enclosure is not sealed to the tailpipe exhaust. The proposed system is based on a partial-flow qualitative sampling system. The enclosure is placed close to the vehicle’s tailpipe to collect representative samples of the exhaust plume when the engine is idling. It is not developed as a total-flow dilution tunnel or a regulatory-level exhaust sampling system. The metal-oxide sensor heads, or MQ-series sensing components, are oriented to engage with the exhaust plume. The ESP8266 microcontroller, buck converter, RTC module, SD card module, and power supply are among the main electronic components installed on the baseboard within a protective enclosure. Instead of using forced inline coupling, the exhaust sampling setup relies on near-field plume exposure. Exhaust gas is not forced through the electronics under pressure, and the enclosure is not sealed to the tailpipe.

The analog outputs of the 8 MQ sensors are connected to an 8 × 1 analog multiplexer (74HC4051) to allow the use of a channel switching line (S0–S2) that is controlled by the ESP-8266 to monitor many exhaust constituents simultaneously, including hydrocarbons, alcohol vapors, methane, LPG, hydrogen, carbon monoxide, and the overall air quality. The single output of the multiplexer is attached to the ADC pin of the microcontroller, and time-division sampling of all gas sensors is possible. The timing of analog-to-digital conversion and multiplexer switching is carefully managed to ensure accurate and stable data acquisition. The ESP8266’s internal ADC completes each conversion in about 200–250 µs. The 74HC4051 multiplexer, controlled by digital select lines (S0–S2), switches channels with negligible delay, but a settling time of 5–10 ms is allowed after each switch to stabilize the sensor output. This delay is crucial for MQ-series sensors due to their high output impedance and transient behavior. As a result, each sensor channel is effectively sampled over 10–11 ms. Scanning all eight sensors takes approximately 80–90 ms, yielding an overall sampling rate of about 10–12 Hz for the entire array. This timing approach ensures reliable signal capture, reduces inter-channel interference, and maintains consistent timing across multiple sensors. The DHT22 sensor is used as an environmental compensation and is connected to a single-wire digital protocol, whereas the correct temporal labelling of sensor values is ensured with the help of the I2C-based RTC module that is connected through SDA and SCL. The microSD card module, via SPI (MISO, MOSI, SCK, CS) offers high speed data recording of long duration field trips, which will guarantee good offline storage. Each component is powered on a common 5 V supply and grounded to ensure constant biasing and stable analog performance. The sensing assembly which includes all the gas sensors is loaded inside a 23-L PVC-made sampling chamber with an exhaust fan that pulls directly the vehicle exhaust at the tailpipe to enable the regulated airflow and even coverage of surfaces of all sensors. The ESP8266 is the heart of the controller, which provides coordination of multiplexer switching, ADC measurements, logs measurements and may transmit measurements wirelessly. A direct sampling of the exhaust gases by a direct extraction technique is done at an intubation point with the tailpipe outlet and the gases are pumped to the sensing chamber with a controlled suction flow. It lacks a mechanism of active dilution on the system. The measurements in this paper are relative changes in concentration in controlled operating conditions as opposed to regulatory grade absolute levels of emissions. The overall design guarantees a multi-gas diagnostic platform, which is portable, low-power, and has a high temporal resolution and long operating range of the resulting real-world vehicular exhaust signatures. Table 1 lists all the gas sensors and other components with their responsivity to various gaseous analytes.

The Drift characterization of the MQ-series gas sensors utilized in the suggested electronic nose system is shown in the table, emphasizing their drift behavior and target gas sensitivities under various environmental circumstances. Table 2 shows how Metal Oxide Semiconductor (MOS) sensors are cross-sensitive and how temperature, humidity, and aging affect sensor stability. It is important to note that MQ-series sensors do not provide selective detection of individual gases such as nitrogen oxides (NOx). Instead, sensors such as MQ135 exhibit broad sensitivity to air quality parameters. Therefore, the proposed system relies on aggregated sensor response patterns rather than explicit gas-wise quantification. The necessity of preprocessing and calibration methods to guarantee dependable pattern recognition performance is supported by this characterization.

### System  Architecture

The proposed system for monitoring vehicle exhaust integrates multiple components, including sensors, controllers, timers, data storage, and power management, into a single, embedded architecture. The MQ-series gas sensor array (MQ2, MQ3, MQ4, MQ6, MQ7, MQ8, MQ9, and MQ135) provides analogue voltage outputs that indicate the presence of combustion-related gases, such as hydrocarbons, hydrogen, methane, LPG, carbon monoxide, and air-quality indicators. The ESP8266 microcontroller only has one ADC input, so an 8 × 1 analog multiplexer (74HC4051) is used to route sensor signals in a time-division manner. The GPIO pins on the ESP8266 power the multiplexer select lines (S0–S2), allowing all sensor channels to be scanned predictably. In the Table 3 “Operational Characteristics and Limitations of MQ-Series Sensors” and Table 4“Gas Targeting and Dynamic Performance Specifications of MQ-Series Sensors.”

The proposed system configuration serves two important purposes:

- Thermal Isolation: The sensors are placed inside a sampling chamber that is physically separated from the vehicle exhaust outlet. As a result, the sensor array is not exposed to the high temperatures of the exhaust stream, and the gas entering the chamber naturally equilibrates to near-ambient temperature before interacting with the sensors.
- Controlled Exposure Environment: The chamber acts as a buffer volume that allows exhaust gases to mix and stabilize before being detected by the gas sensors. This approach ensures consistent and repeatable exposure conditions for the sensing array.

A DHT22 sensor, connected through a digital single-wire protocol, captures environmental variables that significantly impact the performance of a MOS sensor. The I^2^C bus (SDA/SCL) enables a real-time clock (RTC) module to communicate with the computer, assigning an accurate timestamp to each recorded sample. This ensures precise timing in long-term experiments. The SPI interface (MISO, MOSI, SCK, CS) saves all processed data to a microSD memory card. This allows you to collect a large amount of data at once, even when you’re not connected to the internet.

The sensing and electronic circuits are inside a 23-L PVC sampling chamber. The chamber features a 12-V exhaust fan that draws engine exhaust directly from the vehicle’s tailpipe. A buck converter turns the 12-V power supply into a stable 5-V rail that powers all of the electronic modules. To maintain a stable analog reference, all parts share a common ground. Figure 3 depicts the data acquisition setup.

## 5. Dataset Acquisition or Data Description

The embedded data collection system has eight MQ-series gas sensors, a DHT22 temperature-humidity sensor, an I^2^C RTC module, a microSD SPI module, and an 8 × 1 analog multiplexer. The ESP8266 Wi-Fi-enabled microcontroller controls all of these. Each MQ sensor emits an analog signal indicating the concentration of the target gas. The multiplexer enables these signals to be sent to the ESP8266’s ADC one after the other, thereby circumventing the hardware issues. Both the environmental sensing and timestamping unit are running at the same time and all information is recorded and logged at the same time. The power design of the system involves a 12-V battery, and a buck converter to supply all the modules with a constant supply of 5 V. The 23-L PVC chamber has an exhaust fan that maintains the constant exposure of the sensors to engine exhaust. The combined method makes it possible to receive multi-parametric exhaust data, which can be used to conduct a study on vehicle diagnostics.

The MQ-series gas sensors are also preheated around 30 min prior to data collection so that they can work reliably and in a consistent manner. Once the system is stable, it is left to fresh air after 10 min. During this time, 148 baseline samples are taken to create clean reference signals. For exhaust-based measurements, a sealed sampling container with the sensor array is placed close to the exhaust outlets of two- and four-wheeled vehicles. For 10 to 15 min, the exhaust plume from each vehicle is constantly watched. This included engines running on petrol, diesel, and CNG, allowing the researchers to observe distinct chemical signatures from different types of fossil fuels. After each sampling cycle, the container box is opened and aired out for approximately 15 min to eliminate any residual smoke and minimize the impact on subsequent measurements. All recorded data is saved on a memory card connected to the embedded system. We employed this systematic method to create a large, multi-class dataset of vehicular exhaust signatures for machine-learning-based engine classification experiments.

### 5.1. Data Preprocessing and Statistical Analysis

**Figure 4:**
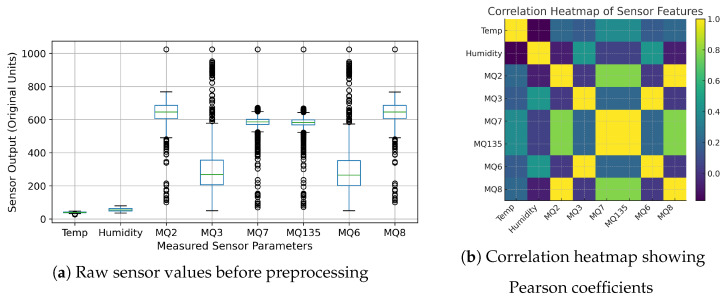
(**a**) Box plot comparison of temperature (°C), relative humidity (%RH), and MQ-series sensor output (original sensor units) before preprocessing, and standardized sensor values (z-score, unitless) across measured sensor parameters. (**b**) Correlation heatmap showing Pearson coefficients among temperature, humidity, and MQ-series gas sensor features. Strong correlations are visible among MQ sensors, indicating synchronized responses under exhaust exposure.

The dataset consists of 3009 observations and 10 variables, collected using a multi-sensor embedded system for modelling vehicular exhaust. The dataset encompasses temperature, humidity, and other responses from MQ-series gas sensors (MQ2, MQ3, MQ4, MQ6, MQ7, MQ135, MQ9, MQ8), along with a categorical target label representing the exhaust source (Fresh Air, Petrol, Diesel, CNG). In our case, we take the emission of vehicles for the pattern recognition in a six-litre container as volume. The dataset contains no missing values in any of its columns, making it immediately suitable for supervised learning, statistical modelling, and pattern-based analyses related to engine classification and exhaust quality assessment. All numerical feature values (temperature, humidity, and sensor outputs from MQ-series sensors) are treated as continuous variables and normalised to z-scores to account for scale differences and prevent any single sensor from driving the learning. The four target classes are then converted to integer labels to facilitate the use of supervised classification models. Figure 4a presents the box plot comparison of the recorded data samples prior to the preprocessing. The dataset is statistically evaluated to know the distributional properties of the continuous features and to test the pairwise relationship among the features and then proceed with modelling. Table 5 gives significant major descriptive statistics (mean, standard deviation, minimum, and maximum) of the temperature, humidity, and the respective responses of the individual MQ-series sensors. Furthermore, we developed a Pearson correlation heatmap in order to further evaluate the relationships between the features, as presented in Figure 4b. The resulting heatmap indicates some significant clusters of correlation of MQ sensors, with respect to the stimulus categories identified in samples.

### 5.2. Correlation Heatmap Interpretation

The correlation heatmap indicates that there are some significant associations between the sensor reactions and environmental factors. The close positive correlations between MQ7 and MQ135 and between MQ2 and MQ8 suggest that these MOS sensors can be sensitive to gases produced by combustion, like carbon monoxide, methane, and other reducing gases found in automobile exhaust. The moderate positive correlations of temperature and some sensor responses indicate that conditions of the environment can affect sensor resistance behavior, whereas humidity shows relatively lower positive correlations, which imply that it has a lesser effect on the responses measured. Although there is some correlated behavior, the sensor array continues to deliver complementary information, which can be used to allow machine learning models to utilize multidimensional response patterns as effective exhaust-gas signature classification.

## 6. Classification Learning Methods

In the classification learning step of the proposed framework, a variety of supervised learning algorithms are applied to capture the complex, non-linear, and cross-sensitive response characteristics produced by the exhaust-gas dataset. MQ-series metal-oxide sensors exhibit overlapping sensitivities to hydrocarbons, alcohol vapours, methane, hydrogen, and other exhaust components, leading to high-dimensional interactions that vary by fuel type. To capture these relationships, the approach uses six classifiers that exhibit complementary behaviours, including linear, kernel-based, neighbourhood-based, and ensemble learning methods. This mixed selection allows evaluating the scale of decision boundaries between simple linear separability and complex hierarchical division and enhanced non-linear transformations. Subsections that follow briefly describe the mathematical bases, decision-making, and implementations behind each of the classifiers.

### 6.1. Logistic Regression (LR)

This paper employs Logistic Regression (LR) as a baseline linear classifier to give an overall measure of performance of more complex models, such as non-linear and ensemble models. The LR is trained to accommodate multiclass, where each of the exhaust classes is assigned a separate logistic model. LR is based on the idea of modelling the conditional probability of the class with the given input feature vector, and the sigmoid function is used with the linear combination of features.

For a given input feature vector $x=(x_{1},x_{2},x_{3},....x_{n})$, LR models the probability that the sample belongs to the positive class y=1 as:

(1) $$P\left(y=1|x\right)=\sigma \left(w^{T}x+b\right)$$

Here, w is the weight vector, b is the bias term, and σ(.) is the sigmoid function given as $\sigma \left(z\right)=\frac{1}{1+e^{-z}}$.

The sigmoid compresses real-valued inputs to the interval (0, 1), allowing the model to output a probability score.

### 6.2. Support Vector Machine (SVM)

A Support Vector Machine (SVM) with a Radial Basis Function (RBF) kernel is used to capture the non-linear overlapping response characteristics of multisensor gas datasets. Aspects of MQ-series sensors exhibit strong cross-sensitivity, in which multiple gases produce correlated changes in sensor resistance, leading to overlapping and complex features that cannot be separated using linear boundaries. The SVM method can effectively address the complexities of multisensor data by using kernel transformations to map input features into a higher-dimensional space, enabling the computation of non-linear decision surfaces that provide maximum separation between classes.

Though SVM is designed for binary classification using a maximum-margin hyperplane to separate two classes. This is extended for multiclass classification using the One-vs-Rest (OvR) strategy. In the RBF-based SVM framework, each binary classifier learns a non-linear decision boundary by implicitly mapping the input *x* into a high-dimensional feature space via the kernel function by evaluating:

(2) $$K\left(x_{i},x_{j}\right)=\exp\left(-\gamma {∥x_{i}-x_{j}∥}^{2}\right)$$

where γ is a kernel parameter that controls the influence of each support vector.

### 6.3. k-Nearest Neighbors (k-NN)

The k-Nearest Neighbours (k-NN) classifier is a non-parametric, instance-based learning approach that predicts class membership based on local neighbourhood structure in the feature space. It assigns class labels based on the proximity of a test sample to the k-nearest training samples, making k-NN appropriate for sensor data when problems naturally cluster into classes. This behaviour is consistent with the MQ-series gas sensors used in this work, which respond to local features in a manner that is coherent (and correlated) with other classes of compounds at different concentrations.

Given a sample *x*, the k-NN algorithm computes the distances between *x* and all training samples $x_{i}$, typically using the Euclidean metric defined as:

(3) $$d\left(x,x_{i}\right)=\sqrt{\sum_{j=1}^{d}{\left(x_{j}-x_{ij}\right)}^{2}}$$

where $d(x,x_{i})$ represents the Euclidean distance between the query sample *x* and a training sample $x_{i}$. The *k* nearest neighbors are then selected, and the predicted class is obtained using majority voting.

### 6.4. Random Forest (RF)

Random Forest classifiers use ensemble learning principles by combining the predictions of many decision trees to achieve highly accurate classification. By training multiple trees on multiple randomly sampled subsets of the data and the individual feature space, RF can alleviate overfitting associated with individual decision trees while improving generalisation under varying exhaust conditions. Each decision tree in the ensemble is trained using bootstrap aggregation (bagging), where a random subset of training samples is selected with replacement, and a random subset of features is chosen at each split. In this study, the RF model is specified with 400 trees and class-balanced weighting to suite the moderately imbalanced dataset. At each split node, a random subset of features m⊂d is selected, and the optimal split is determined using the Gini impurity:

(4) $$G=1-\sum_{k=1}^{K}p_{k}^{2}$$

where $p_{k}$ represents the proportion of samples belonging to class *k*.

The final prediction of the Random Forest classifier is obtained using majority voting across all *T* trees:

(5) $$\hat{y}=\mathrm{mode}{\left\{h_{t}\left(x\right)\right\}}_{t=1}^{T}$$

where T=400 in this study.

### 6.5. Gradient Boosting Decision Trees (GBDT)

Gradient Boosting Decision Trees (GBDT) is an ensemble learning method that builds a sequence of decision trees. Each tree, in turn, attempts to compensate for the error of the previous trees by fitting to the negative gradients (i.e., the residuals) of the loss function. In contrast to Random Forest, which builds multiple trees independently using bagging, GBDT builds trees in an additive, stage-wise fashion. This highly effective approach capitalises on a tree’s ability to capture complex feature interactions and non-linear patterns observed in multisensor exhaust readings. MQ-series sensors often produce gases with overlapping and cross-sensitive response signals, and GBDT’s sequential and iterative refinement procedure enables the model to learn these challenging boundaries. Gradient Boosting Decision Trees (GBDT) constructs an additive model by sequentially combining weak learners (decision trees). The model is defined as:

(6) $$F\left(x\right)=\sum_{t=1}^{T}\alpha_{t}h_{t}\left(x\right)$$

where $h_{t}\left(x\right)$ represents the decision tree at iteration *t*, and $\alpha_{t}$ is the corresponding weight.

The model is trained by minimizing a loss function:

(7) $$L=\sum_{i=1}^{N}L\left(y_{i},F\left(x_{i}\right)\right)$$

At each iteration, the new tree is fitted to the negative gradient (residuals) of the loss function:

(8) $$r_{i}^{\left(t\right)}=-{\left[\frac{\partial L(y_{i},F\left(x_{i}\right))}{\partial F(x_{i})}\right]}_{F=F_{t-1}}$$

The model is updated in a stage-wise manner as:

(9) $$F_{t}\left(x\right)=F_{t-1}\left(x\right)+\eta \;h_{t}\left(x\right)$$

where η is the learning rate controlling the contribution of each tree.

This gradient-based optimization enables the model to iteratively reduce classification error by focusing on hard-to-classify exhaust patterns.

### 6.6. Extreme Gradient Boosting (XGBoost)

Extreme Gradient Boosting (XGBoost) is a popular advanced ensemble-learning technique that builds on a general gradient boosting framework with additional optimised regularisation, parallelised tree construction, and enhanced methods for handling sparse and noisy data. These characteristics indicate that XGBoost is particularly useful for modelling the non-linear, cross-sensitive, and partially correlated response patterns of MQ-series gas sensors under different exhaust conditions. It is essential to understand that XGBoost builds an additive model from multiple decision trees, where each tree further reduces the ensemble’s residual errors while optimising model complexity to control overfitting. Extreme Gradient Boosting (XGBoost) extends gradient boosting by incorporating regularization and second-order optimization. The model is defined as an additive ensemble of decision trees:

(10) $${\hat{y}}_{i}=\sum_{t=1}^{T}f_{t}\left(x_{i}\right),\;f_{t}\in F$$

where $f_{t}\left(x\right)$ represents the decision tree at iteration *t*.

The objective function is defined as:

(11) $$L^{\left(t\right)}=\sum_{i=1}^{N}l\left(y_{i},{\hat{y}}_{i}^{\left(t\right)}\right)+\sum_{t=1}^{T}\Omega \left(f_{t}\right)$$

where l(·) is the loss function and $\Omega (f_{t})$ is the regularization term given by:

(12) $$\Omega \left(f\right)=\gamma T+\frac{1}{2}\lambda {∥w∥}^{2}$$

To optimize the objective, XGBoost uses a second-order Taylor expansion:

(13) $$L^{\left(t\right)}\approx \sum_{i=1}^{N}\left[g_{i}f_{t}\left(x_{i}\right)+\frac{1}{2}h_{i}f_{t}^{2}\left(x_{i}\right)\right]+\Omega \left(f_{t}\right)$$

where $g_{i}=\frac{\partial l}{\partial {\hat{y}}_{i}^{\left(t-1\right)}}$ and $h_{i}=\frac{\partial^{2}l}{\partial {\hat{y}}_{i}^{2(t-1)}}$ represent the first and second-order gradients, respectively.

The inclusion of second-order gradient information and regularization enables XGBoost to achieve superior performance and robustness, particularly for noisy and cross-sensitive multisensor datasets.

## 7. Results and Discussion

This section outlines the experimental work conducted on the multi-sensor exhaust-gas dataset and describes the comparative behaviour of the models studied across different experimental settings. This section is divided into two subsections. The first subsection presents the full experimental setup, including preprocessing steps, classification models, hyperparameter selection, and the evaluation parameters used to measure performance. The second subsection presents the results across the different experimental settings.

The experimental workflow has been established to ensure that every classifier being applied to the multi-sensor exhaust-gas dataset receives a fair and consistent assessment throughout the evaluation. The undertaking of the experiment is separated into the preprocessing methodology, the selection and setup of classification models, the data-enhancement strategy and validation practices used to measure model generalisation. The implementation of experimentation itself is conducted in Python 3.12 The dataset contains measurements of temperature, humidity, and eight gas sensors that provide analog readings taken from cross-selective MQ-series sensors, meaning the features all consist of continuous variables. They are prepared by standardizing the features with z-score normalization through the StandardScaler transformer so that every feature does not potentially dominate results with the learning.

The target classes are labelled as Fresh Air, PETROL, DIESEL, and CNG, encoded into integer categories, so that learning models can be utilised on the response. An 80:20 split has been used for the training/testing sets. During experimentation, an evaluation of six supervised classifiers is conducted to provide a range of decision boundaries and learning curves, including LR, SVM with RBF kernels, k-NN, RF, GBDT, and XGBoost. Various classifier models are also utilised, including linear models (LR), kernel-based methods (SVM), neighbourhood classifiers (k-NN), and ensemble learning (RF, GBDT, XG Boost). These two aspects, all classifiers and differences in model types, are implemented as variables in the evaluation task to allow for a wide net to capture a variety of elemental modelling behaviours related to non-linear and cross-sensitive sensor responses.

**Figure 5:**
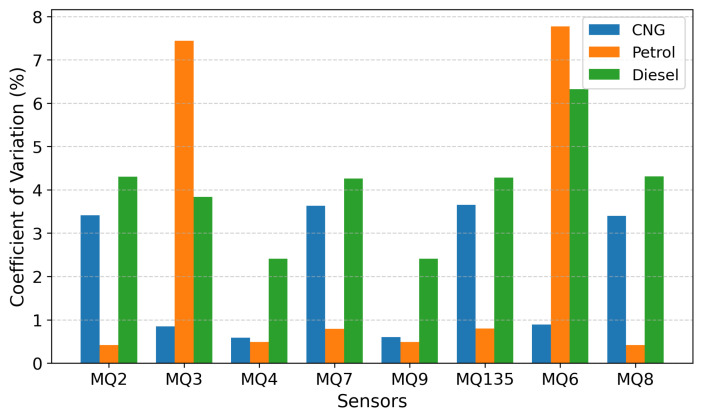
Repeatability Comparison Across Fuel Types.

To ensure that the sensor data represents consistent and reliable physical measurements, a repeatability analysis is conducted across multiple independent acquisition sessions. Repeatability is a fundamental requirement in sensor-based systems, as it verifies that the sensing platform produces stable outputs under identical operating conditions and is not influenced by random environmental fluctuations or measurement inconsistencies. In the proposed study, repeatability is evaluated by organizing the collected data into session-wise groups corresponding to repeated measurements for the same vehicle and fuel type. For each sensor, the mean response is computed within each session, followed by the estimation of the standard deviation across sessions. The coefficient of variation (CV), defined as the ratio of the standard deviation to the mean response is used as the primary metric for assessing repeatability. Figure 5 shows the percentage CV achieved across different fuel type for each gas sensor.

Under CNG operation, all sensors exhibit excellent repeatability characteristics. The coefficient of variation remains consistently below 5% for all sensors, with several sensors such as MQ3 (0.85%), MQ4 (0.59%), MQ9 (0.60%), and MQ6 (0.89%) demonstrating extremely low variability. For Petrol-based emissions, the majority of sensors continue to exhibit strong repeatability, with MQ2, MQ4, MQ7, MQ9, MQ135, and MQ8 all maintaining CV values below 1%. This indicates highly consistent sensor behavior across sessions. However, sensors MQ3 and MQ6 exhibit significantly higher variability, with CV values of approximately 7%. A similar trend is observed under Diesel conditions. Most sensors, including MQ2, MQ4, MQ7, MQ9, MQ135, and MQ8, demonstrate stable repeatability with CV values below 5%. In contrast, MQ3 and MQ6 again exhibit higher variability consistent with their sensitivity to hydrocarbon-rich exhaust components. To further examine the temporal behavior of the sensing system, a drift analysis is performed using time-ordered sensor data. MOS sensors are known to exhibit inherent response dynamics, including an initial transient phase followed by a stabilization region during continuous exposure to gases. All sensors demonstrate a clear transition from an initial transient response to a relatively stable steady-state region. Within this steady-state phase, the sensor outputs exhibit smooth and gradual variations over time without abrupt fluctuations or random spikes. Although, MQ3 and MQ6, show comparatively slower stabilization and extended response dynamics.

The hyperparameters used for each classifier are finalised through an iterative process, and the finalised settings for each experiment are summarised in Table 6. The LR is configured with a larger max iteration limit and class-balanced weighting to guarantee proper convergence on the imbalanced dataset. The SVM is implemented with an RBF kernel, probability estimation, and balanced class weighting to capture non-linear patterns in their sensor data. The k–NN classifier is examined with seven neighbours to provide a locally sensitive and smooth decision surface. The RF implemented 400 trees, using class balancing to capture heterogeneity within the ensemble without increasing overall variance. The GBDT classifier used the default boosting parameters (and a random seed to ensure replicability). The main XGBoost model also used default settings, with 600 estimators, a maximum depth of 4, a learning rate of 0.05, and subsampling and column sampling set to 0.9, plus L2 regularisation. These hyperparameter settings are used to capture the exhaust-gas data well, facilitating the proposed experiments.

The multi-sensor exhaust-gas dataset has a slight class imbalance, where samples of the Fresh Air and Diesel class are relatively lower when compared to the Petrol and CNG classes. To help relieve the class imbalance issue, the authors use a technique entitled SMOTE to create synthetic data points in the feature space of the minority classes and improve the representation of class-boundaries. This research study employs SMOTE only on the training subset, using five nearest neighbors for the training the model, while also ensuring that the test set remains untouched and free from manually created samples. In addition to the single train-test split, the experiments also used five-fold stratified cross-validation. Stratification ensures that each fold maintains the original proportions of the classes, which is necessary for imbalanced-sensitive datasets. For each fold, four parts of the data are used for training, with one part used for validation, and this continues until each fold has been utilised once as validation. The results from the five folds are averaged to provide stable estimates of accuracy and F-1 scores for the models. This result provided a more reliable performance measure across different data folds and reduced the variance associated with a single hold-out evaluation.

Various evaluation metrics are used to evaluate the classifier’s performance. A confusion matrix is a basic tool for evaluating classification performance across all target classes. This study has four target classes, so a 4×4 matrix is used to count the number of predictions for each proper–predicted class pairing. The evaluation metrics are determined based on the following measures:

- Accuracy (A): Accuracy measures the proportion of correctly classified instances across all classes.
- Precision (P): Precision quantifies how many predicted samples of a class are actually correct.
- Recall (R): Recall measures how effectively the model identifies samples belonging to a class.
- F1Score ($F_{1})$: Represents the harmonic mean of precision and recall, providing a balanced assessment of predictive capability.

(14) $$A=\frac{\left((TP)+TN\right)}{\left((TP)+TN)+(FP+FN\right)}$$

(15) $$P=\frac{\left(TP\right)}{\left(TP)+(FP\right)}$$

(16) $$R=\frac{\left(TP\right)}{\left(TP)+(FN\right)}$$

(17) $$F_{1}=\frac{2\times (P\times R)}{\left(P+R\right)}$$

**Figure 6:**
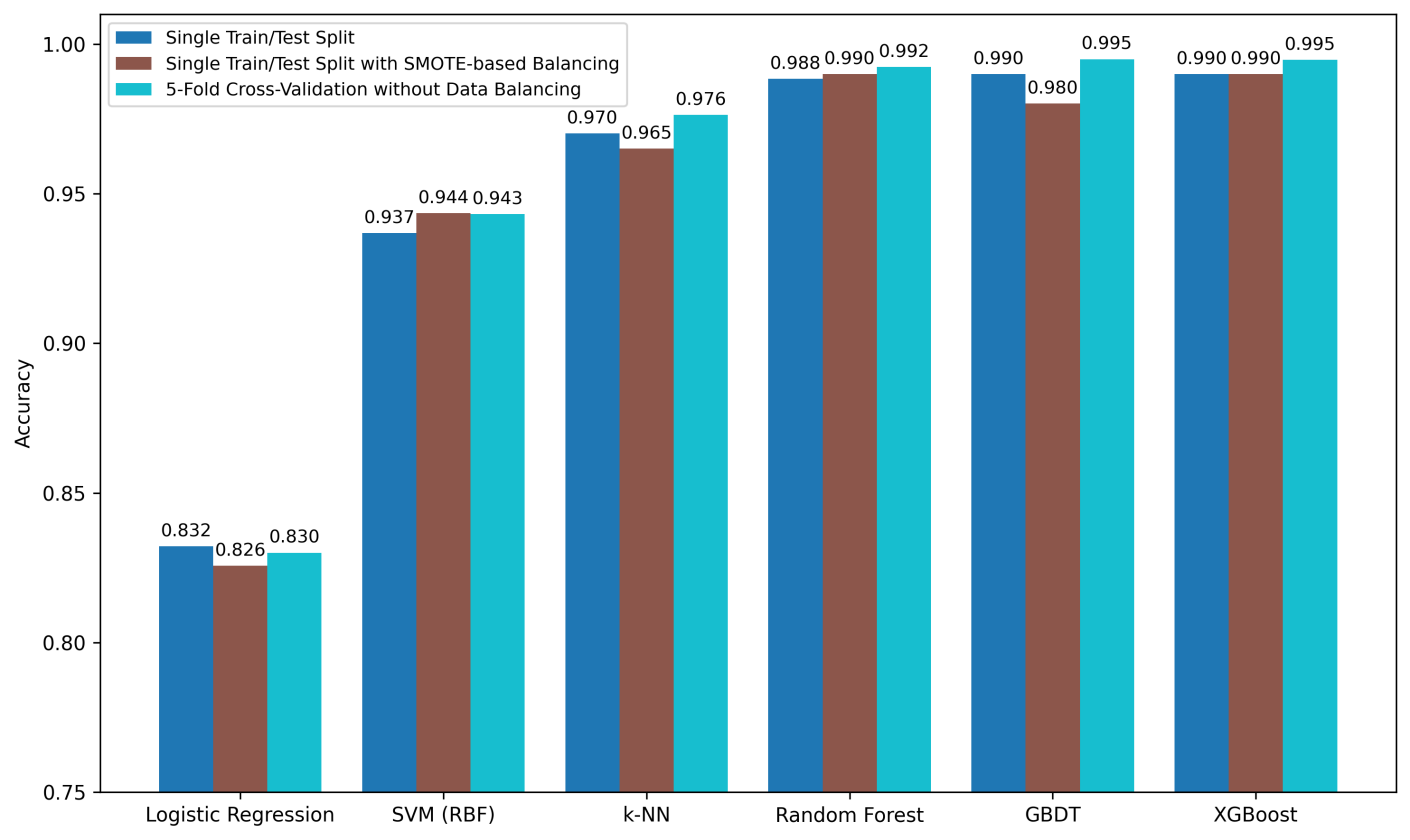
Comparison of model accuracy under various machine learning models and different experimental pipelines.

**Figure 7:**
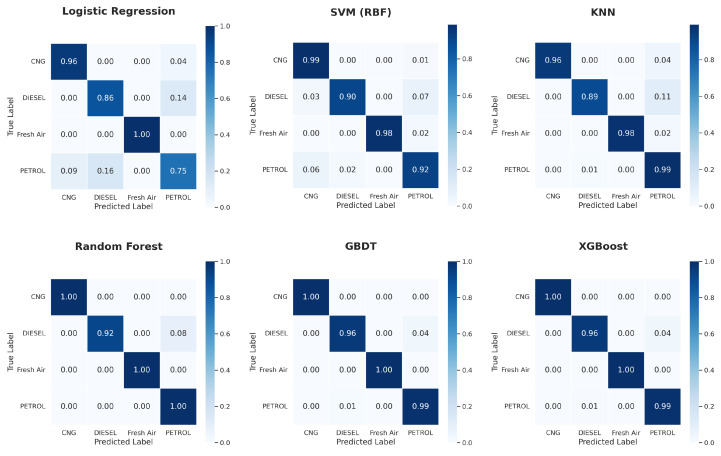
Confusion matrices of all classifiers with a single train-test split.

**Figure 8:**
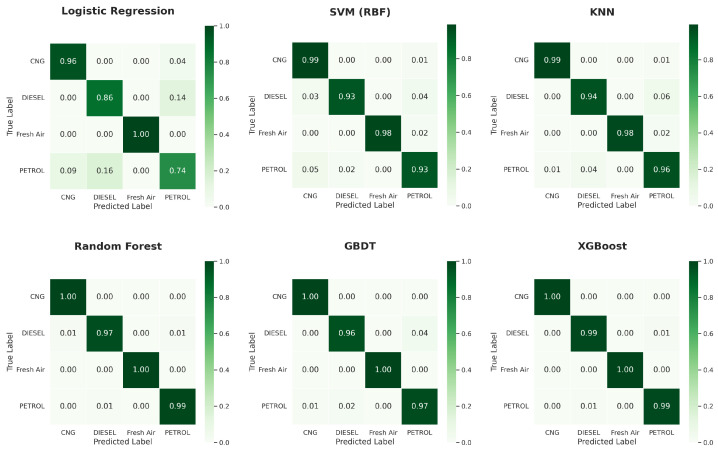
Confusion matrices of all classifiers under a single train-test split with SMOTE-based balancing.

**Figure 9:**
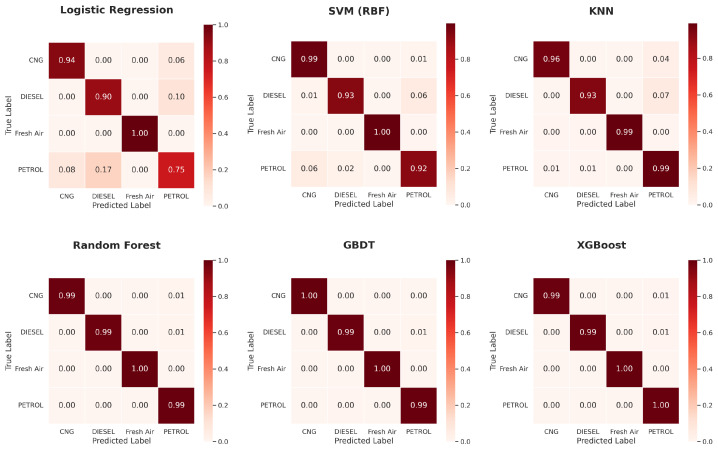
Confusion matrices of all classifiers under five-fold cross-validation without data balancing.

Figure 6 shows a comparative performance of all the classifiers under different experimentation settings. From the results, it is evident that all three ensemble methods outperform other classifiers and achieve the best results. Figure 7, Figure 8 and Figure 9 show the confusion matrix obtained across different configurations.

The confusion matrices presented for all three configurations demonstrate a consistent pattern of classifier performance. The small number of misclassifications observed primarily occur between petrol and diesel samples. From a physicochemical perspective, this behaviour is expected because both combustion processes produce overlapping mixtures of hydrocarbons, carbon monoxide, and partially oxidised species. In the simple single train-test, the linear LR misclassified petrol samples as diesel or CNG quite often, showing the incapacity of simple linear decision boundaries to distinguish among the overlapping signatures of the exhaust samples interpreted by MQ sensors. The non-linear mapping to the kernel made much improvement for the SVM model, but given the similarity and interchangeability of some diesel and petrol samples, not all diesel and petrol are distinguished. The k-NN model is even better comparatively. The ensemble methods achieved extremely high diagonal accuracy with no or extremely few off-class predictions, which represents stable decision boundaries that can capture the more nuanced non-linear relationships along temperature, humidity, and a gas-sensor data profile. In addition, recall is improved for the minority classes, specifically for Fresh Air and CNG classes, with the implementation of SMOTE, particularly when using kernel and distance-based models.

Table 7 provides a summary of all six classifiers’ performance under the selected condition of a single stratified 80:20 train-test split, without any data-balancing technique applied to split the dataset. This experiment provides a detailed interpretation of the variation in performance of the different linear, kernel-based, nearest neighbor-based, and ensemble-learning-based approaches when presented with the classification of multi-sensor exhaust-gas data. The performance of the LR model is 0.8322, which exemplifies the limitation of linear decision boundaries to distinguish between the non-linear and cross-sensitive pattern of response of the MQ-series sensor. The SVM with an RBF kernel produces an improvement of 0.9369 in performance accuracy and a 0.9375 performance macro F1-score. This improvement confirms the performance and advantages of the suitability of a non-linear kernel. The k-Nearest Neighbors classifier yields a larger improvement in performance accuracy (0.9701), providing a strong relative performance indication that the local similarity measures can adequately capture a reasonably accurate neighborhood-level relationship of sensor readings to the mapping. Finally, all ensemble models (RF, GBDT, and XGBoost) perform the best, with Random Forest achieving an accuracy of 0.9884, followed by GBDT and XGBoost, achieving an accuracy of 0.9900 with identical precision, recall, and F1-score performance.

In the provided Table 8, all classifiers trained with SMOTE are reported; this allows for evaluation of the impact class distribution influence has on learning the model, especially for the classes Fresh Air and DIESEL, both of which had less data available at the start of analyses. All classifiers show noticeable, but not extreme, impact of SMOTE on learning the LR model; the accuracy reported is 0.8256. The RBF kernel with SVM exhibited some measurable improvement; accuracy is reported to as 0.9435. The k-NN classifier showed similar improvements; accuracy is reported to improved to 0.9651; which illustrates interested neighbor consistency. The set of classifiers belong to ensemble-militaristic learning at judges continue to express the best performance, as noted in cheer configuration area. The RF models accuracy is reported to be 0.9900 and XGBoost achieved the same accuracy of 0.9900. GBDT dropped correspondingly a reported accuracy of 0.9900 to drop 0.9801.

Table 9 reports the performance of all classifiers under five-fold stratified cross-validation without applying any class-balancing technique. This technique provides more reliable results of classifiers across multiple train–validation partitions. LR continues to show limited performance, achieving an accuracy of 0.8300. SVM with an RBF kernel achieves an overall accuracy of 0.9432, while K-NN achieves 0.9764. All the ensemble methods achieve accuracies of 0.9924, 0.9950, and 0.9947 under RF, GBDT, and XGBoost, respectively.

Moreover, the results of Table 10 summarize the mean and standard deviation of accuracy, macro-F1, and weighted-F1 across five stratified folds. The results demonstrate very low performance variance across folds for all models. Specifically for ensemble methods, the standard deviation remains below 0.004. This confirms that the high accuracies reported are not due to favorable data partitioning or overfitting but rather reflect consistent model generalization. Moreover, the trends of stability are similar in all the experimental settings, which also confirms consistency. Those findings will be considered the statistical data that prove the effectiveness of the suggested multi-sensor exhaust classification framework.

**Figure 10:**
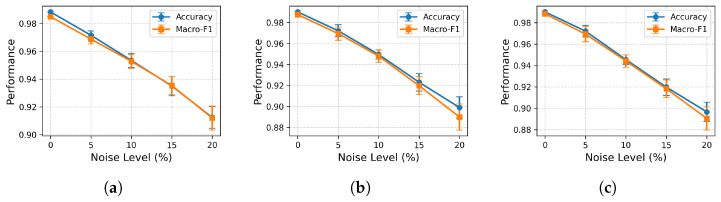
Impact of Noise on Classification Performance (**a**) Random Forest, (**b**) Gradient Boosting Decision Trees (GBDT), (**c**) Extreme Gradient Boosting (XGBoost).

While cross-validation results demonstrate strong classification performance under standard conditions, it is equally important to evaluate the robustness of the proposed framework under realistic sensor perturbations. In practical deployments, gas sensor readings are often affected by measurement noise, environmental fluctuations, and minor hardware inconsistencies. To simulate such conditions, a noise injection experiment is also conducted. Gaussian noise was synthetically added to the sensor features at varying levels of 0%, 5%, 10%, 15%, and 20% of the feature standard deviation. The classifier models are then evaluated on on the perturbed test data. Figure 10 shows the performance as model accuracy with increasing noise level in ensemble models. RF shows high baseline performance with 98.84%, but with noise, it shows a slightly sharper decline in accuracy. At 10% noise, the model still maintains an accuracy of 96.35%, and even at 20% noise, it achieves 89.20% accuracy. This behavior suggests that RF is highly accurate under clean conditions; its boosted structure makes it somewhat more sensitive to accumulated noise in feature space. XGBoost model demonstrates strong robustness to noise. XGBoost achieves the highest baseline performance with an accuracy of 99.00%. At 10% noise, accuracy remains high at 95.34%, but at 20% noise, it reduces to 90.32%. GBDT exhibits a balanced performance profile, with a baseline accuracy of 99.00%. Its degradation trend is smoother than RF and comparable to XGBoost. At 20% noise, it maintains an accuracy of 91.02%. Among the evaluated models, GBDT exhibit superior robustness to noise, maintaining more stable performance at higher perturbation levels.

## 8. Discussion and Limitation

However, it is important to note that the specified system is to be designed in a manner that will allow categorizing the patterns of exhaust-gas signature on a qualitative scale but not to quantify emissions on a calibration or quantitative one. The observed performance is thus an indication of the separability of the exhaust-response patterns in the collected data and cannot be interpreted as predictive capability of all vehicle types and operating conditions. Although the suggested framework is successful in classification, some weaknesses have to be admitted. The controlled sampling chamber and a restricted number of vehicle types are used to collect the dataset, and they might not explore the diversity of the real-world driving conditions. Besides, MQ-series sensors are cross-sensitive and can be affected by long-term environmental drift and hence may impact the performance of generalization when used in the field over a long duration. The non-specificity of MQ sensors may limit their use for distinguishing gases in changing environments. Though the 5-fold cross-validation is employed, it is still possible that the random partitioning could add a sample of the same vehicle or the same acquisition session in both the training set and the testing set. Moreover, the current research lacks sensitivity of the sensor array to laboratory-grade reference measurements (e.g., NDIR or FTIR analyzers). Subsequently, the measured signals can be regarded as relative response patterns and not absolute gas concentrations. Long-term stability and measurement reproducibility can also be affected by sensor aging, a baseline shift, and environmental factors like changes in temperature and humidity. The next stage of work will address these limitations with the help of massive data collections of real-life data and operating conditions of various classes of vehicles. The identification of vehicles and sampling-session metadata will also be explicitly registered in future work to facilitate grouped validation procedures like Group KFold and leave-one-vehicle-out validation.

## 9. Conclusions and Future Work

This study demonstrates that a cheap embedded multi-sensor gas framework with machine learning classifiers can classify the state of vehicle exhaust with a high degree of reliability in terms of Fresh Air, PETROL, DIESEL, and CNG. The paper has utilized MQ series gas sensors with environmental sensing, temporal indexing, and systematic pre-processors because the technique proposed measures realistic exhaust signatures that are indicative of a vehicle being operated on the road. It was experimented that ensemble-based models, including GBDT and XGBoost models, showed high performance and provided a significant superiority over linear and kernel-based models. The results demonstrate that the non-linear, cross-sensitive behavior characteristic of the MOS sensor arrays can be well modeled by enriching the architectures.

Besides, future experiments will explore sensor calibration methods based on reference-grade analyzers and introduce drift-compensation methods to enhance the long-term stability of measurements. Better sampling structures such as controlled inlet flow paths and metallic sampling enclosures will also be considered to mitigate the environmental interference and improve the consistency of the measurements. Moreover, it will be investigated how to be integrated with edge-based real-time processing architectures so that on-road deployment can be scaled. The framework can be applied to the hybrid and electric powertrain setting, and deep learning models, including LSTM to model temporal exhaust patterns, can also be used to enhance the robustness of the system in real-world dynamic settings. The last task to be conducted in the future concerns the cross-vehicle validation and domain-adaptation approaches to assess the generalization of the proposed solution to various vehicle models, fuel properties, and the environment. We will also later in the future expand the current sensing framework to cover the monitoring of the particulate matter with the help of standardized techniques of sampling like dilution-based sampling of particles.

## Figures and Tables

**Table 1:** Gas sensor elements and their responsivity to various gaseous analytes.

Component	Function
MQ2	Smoke
MQ3	Alcohol
MQ4	Methane
MQ6	LPG
MQ7	CO
MQ8	Hydrogen
MQ9	CO/HC
MQ135	Air Quality
DHT22	Temp/Humidity
RTC	Timestamp
SD Card	Storage
Multiplexer	ADC Expansion
ESP8266	Controller

**Table 2:** Drift Characterization of MQ-Series Gas Sensors Used in the Electronic Nose System.

Sensor	Target Gas/Sensitivity	Drift Characteristics
MQ2	LPG, Hydrogen, Methane, Smoke	Moderate drift due to temperature and humidity variations
MQ3	Alcohol (Ethanol vapors)	Low drift when heater voltage remains stable
MQ4	Methane (CH_4_), Natural Gas	Moderate drift influenced by humidity
MQ6	LPG, Butane	Moderate drift during long-term hydrocarbon exposure
MQ7	Carbon Monoxide (CO)	Drift due to cyclic heater operation
MQ8	Hydrogen (H_2_)	Moderate drift affected by ambient humidity
MQ9	Carbon Monoxide (CO), Methane, LPG	Drift caused by temperature cycling and sensor aging
MQ135	NH_3_, NO_x_, Benzene, Smoke, CO_2_	Higher drift due to cross-sensitivity and environmental conditions

**Table 3:** Operational Characteristics and Limitations of MQ-Series Sensors.

Sensor	Sensitivity Characteristics	Drift Behavior	Environmental Dependence	Key Limitations
MQ2	High sensitivity to combustible gases	Baseline drift over time	Strong temperature and humidity dependence	Broad cross sensitivity
MQ3	High alcohol vapor sensitivity	Moderate long-term drift	Humidity affects baseline resistance	Sensitive to other VOCs
MQ4	Enhanced methane response	Moderate resistance drift	Heater temperature sensitive	Limited discrimination in mixed gases
MQ6	Strong LPG response	Baseline shifts with aging	Humidity dependent	Overlapping response with MQ2
MQ7	Optimized for CO with cyclic heating	Noticeable drift without recalibration	Strong temperature dependence	Cross-sensitive to H_2_
MQ8	High hydrogen sensitivity	Long-term resistance drift	Sensitive to humidity changes	Responds to other reducing gases
MQ9	Dual sensitivity (CO + LPG)	Baseline resistance drift	Sensitive to heater cycling	Limited gas selectivity
MQ135	Broad air-quality response	Moderate long-term drift	Highly humidity-dependent	Very broad cross-sensitivity

**Table 4:** Technical Characterization of MQ-Series Gas Sensors Used in the Electronic Nose System.

Sensor	Primary Target Gas(es)/Sensitivity	Typical Detection Range (ppm)	Response Time (T90)	Recovery Time
MQ2	LPG, CH_4_, smoke, H_2_	300–10,000	<10 s	<30 s
MQ3	Alcohol vapors (ethanol)	25–500	<10 s	<30 s
MQ4	Methane (CH_4_)	300–10,000	<10 s	<30 s
MQ6	LPG, propane, butane	200–10,000	<10 s	<30 s
MQ7	Carbon Monoxide (CO)	20–2000	<60 s	<90 s
MQ8	Hydrogen (H_2_)	100–10,000	<10 s	<30 s
MQ9	CO and combustible gases	10–10,000	<10–60 s	<60 s
MQ135	NH_3_, NO_x_, benzene, VOCs	10–1000	<10 s	<30 s

**Table 5:** Statistical Summary of Continuous Features.

Feature	Unit	Mean	Std	Min	Max
Temperature	°C	27.18	1.35	24.90	31.20
Humidity	%	51.66	3.95	45.10	59.80
MQ2	ADC value	620.23	198.21	341	1404
MQ3	ADC value	214.87	63.12	100	402
MQ7	ADC value	590.42	204.73	331	1503
MQ135	ADC value	583.72	209.41	330	1469
MQ6	ADC value	214.33	63.04	102	403
MQ8	ADC value	620.38	198.54	344	1409

**Table 6:** Hyper-parameters and other experimentation settings.

Model	Key Hyper-Parameters
LR	max_iter = 2000, multi_class = “auto”, class_weight = “balanced”, random_state = rng
SVM (RBF)	kernel = “rbf”, probability = True, class_weight = “balanced”, random_state = rng
k-NN	n_neighbors = 7
RF	n_estimators = 400, random_state = rng, class_weight = “balanced”
GBDT	random_state = 42
XGBoost	n_estimators = 600, max_depth = 4, learning_rate = 0.05, random_state = 42

**Table 7:** Performance of all classifiers under a single train-test split.

S.No.	Model	Accuracy	Precision	Recall	F1-Score
1	LR	0.8322	0.8692	0.8322	0.8382
2	SVM (RBF)	0.9369	0.9411	0.9369	0.9375
3	k-NN	0.9701	0.9706	0.9701	0.9699
4	Random Forest	0.9884	0.9884	0.9884	0.9882
5	GBDT	0.9900	0.9900	0.9900	0.9900
6	XGBoost	0.9900	0.9900	0.9900	0.9900

**Table 8:** Performance of all classifiers under a single train-test split with SMOTE-based balancing.

S.No.	Model	Accuracy	Precision	Recall	F1-Score
1	LR	0.825	0.863	0.825	0.831
2	SVM (RBF)	0.943	0.947	0.943	0.944
3	k-NN	0.965	0.967	0.965	0.965
4	Random Forest	0.990	0.990	0.990	0.990
5	GBDT	0.980	0.980	0.980	0.980
6	XGBoost	0.990	0.990	0.990	0.990

**Table 9:** Average performance of all classifiers under five-fold cross-validation without data balancing.

S.No.	Model	Accuracy	Precision	Recall	F1-Score
1	LR	0.8300	0.8687	0.8302	0.8368
2	SVM (RBF)	0.9432	0.9470	0.9432	0.9437
3	k-NN	0.9764	0.9765	0.9764	0.9763
4	Random Forest	0.9924	0.9924	0.9924	0.9924
5	GBDT	0.9950	0.9950	0.9950	0.9950
6	XGBoost	0.9947	0.9947	0.9947	0.9947

**Table 10:** Five-Fold Cross-Validation Stability Analysis.

S.No.	Model	Accuracy (Mean ± Std)	Macro-F1 (Mean ± Std)	Weighted-F1 (Mean ± Std)
1	Logistic Regression	0.8302 ± 0.0051	0.8440 ± 0.0042	0.8369 ± 0.0045
2	SVM (RBF)	0.9432 ± 0.0027	0.9461 ± 0.0040	0.9437 ± 0.0027
3	k-NN	0.9764 ± 0.0083	0.9747 ± 0.0086	0.9763 ± 0.0084
4	Random Forest	0.9924 ± 0.0040	0.9923 ± 0.0041	0.9924 ± 0.0040
5	GBDT	0.9950 ± 0.0024	0.9946 ± 0.0027	0.9950 ± 0.0023
6	XGBoost	0.9947 ± 0.0039	0.9941 ± 0.0043	0.9947 ± 0.0038

## Data Availability

The data supporting the findings of this investigation are accessible from the corresponding author upon reasonable request.

## Abbreviations

The following abbreviations are used in this manuscript:

ML	Machine Learning
LR	Logistic Regression
K-NN	k-Nearest Neighbors
SVM	Support Vector machine
GBDT	Gradient Boosting Decision Trees
RF	Random Forest
SMOTE	Synthetic Minority Oversampling Technique
ADC	Analog to Digital Converters
CO	Carbon Monoxide
PEMS	Portable Emission Measurement Systems
PM	Particulate Matter
HC	Hydro Carbon
CNG	Compressed Natural Gas
NDIR	Non-Dispersive Infrared
FTIR	Fourier Transform Infrared
XGBoost	Extreme Gradient Boosting
VOCs	Volatile Organic Compounds
RH	Relative Humidity
